# Supplementary material for: Stacking Model‐Based Classifiers for Dealing With Multiple Sets of Noisy Labels
Source: Biom J. 2025 Mar 12;67(2):e70042. doi: 10.1002/bimj.70042 (PMC11898607; doi:10.1002/bimj.70042)
Supplement: Supplementary file 1 — Supporting Information [file BIMJ-67-e70042-s001.zip › density-based-ensemble-model-main/competitors_real_data/netlab-master/netlab-master/help/demhint.htm]

Netlab Reference Manual demhint


# demhint

## Purpose

Demonstration of Hinton diagram for 2-layer feed-forward network.

## Synopsis

```
demhint
demhint(nin, nhidden, nout)
```

## Description

`demhint` plots a Hinton diagram for a 2-layer feedforward network
with 5 inputs, 4 hidden units and 3 outputs. The weight vector is
chosen from a Gaussian distribution as described under `mlp`.

`demhint(nin, nhidden, nout)` allows the user to specify the
number of inputs, hidden units and outputs.

## See Also

`hinton`, `hintmat`, `mlp`, `mlppak`, `mlpunpak`

---

**Pages:**
Index


---

Copyright (c) Ian T Nabney (1996-9)
